# Antioxidant Mechanism is Involved in the Gastroprotective Effects of Ozonized Sunflower Oil in Ethanol-Induced Ulcers in Rats

**DOI:** 10.1155/2007/65873

**Published:** 2007-01-18

**Authors:** Zullyt B. Zamora Rodríguez, Ricardo González Álvarez, Dailén Guanche, Nelson Merino, Frank Hernández Rosales, Silvia Menéndez Cepero, Yaima Alonso González, Siegfried Schulz

**Affiliations:** ^1^Laboratory of Biological Assays, Department of Biomedical Research, Ozone Research Center, P.O. Box 6414, Havana, Cuba; ^2^Laboratory of Pathological Anatomy, Department of Biomedical Research, Center of Pharmaceutical Chemistry, P.O. Box 16042, Havana, Cuba; ^3^Veterinary Services and Laboratory Animal Medicine, Philipps University of Marburg, 35033 Marburg, Germany

## Abstract

This research was performed in order to determine the potential protective effects of ozonized sunflower oil (OSO) in the injury of rat gastric mucosa induced by absolute ethanol and as well as to elucidate the role of reactive oxygen species (ROS), lipid peroxidation, and some important constituents of antioxidant defense such as superoxide dismutase (SOD), glutathione peroxidase (GSH-Px), and catalase (CAT) in these effects. OSO was administered to rats intragastrically by a cannula and it was applied during four days to animals. The doses of OSO administered daily to each group of rats were 4, 12, and 24 mg/kg, respectively, and one hour after the last treatment, absolute ethanol (1 mL/200 mg body weight) was administered. Our results showed that gastric ulcer index was significantly reduced in rats pretreated with OSO as compared with ethanol-treated controls. However, in rats pretreated with OSO, no significant reduction of TBARS content in gastric mucosa was found as compared to those rats treated with ethanol alone. In contrast, SOD and GSH-Px activities were significantly increased in gastric mucosa of OSO-pretreated rats with respect to those treated with ethanol alone. In summary, our results demonstrate that OSO pretreatment exerts protective effects in ethanol-induced gastric ulcers in rats. Furthermore, these results provide evidence that these protective effects of OSO are mediated at least partially by stimulation of some important antioxidant enzymes such as SOD and GSH-Px, which are scavengers of ROS and therefore prevent gastric injury induced by them.

## 1. INTRODUCTION

Ozonized sunflower oil (OSO) for oral application is a registered drug that is obtained from the reaction between ozone and sunflower oil under appropriate conditions according to a process developed in our center.

OSO has shown antimicrobial effects against virus, bacteria, and fungi [1]. In addition, preclinical toxicological studies with OSO have demonstrated that this drug is safe and not genotoxic [2], whereas in clinical trials Phase II and Phase III have reported very few and no severe adverse reactions in patients.

On the other hand, the use of OSO in the treatment of *Giardia lamblia* infection has been studied in animal models and humans by oral administrations which have demonstrated the therapeutic effectiveness of OSO in this disease [3].

The lipid peroxidation mediated by reactive oxygen species (ROS) is an important cause of destruction and damage to cell membranes and it is involved in the pathogenesis of acute mucosal injury induced by ethanol, ischemia-reperfusion, and indomethacin [4, 5]. In addition, Glutathione (GSH) is an important constituent of intracellular protective mechanism against a number of noxious stimuli, and it is known as a major low molecular weight scavenger of free radicals in cytoplasm. Sulphydryl (SH) containing compounds, and also agents that modify SH groups, prevents the acute hemorrhagic erosions caused by ethanol, nonsteroidal anti-inflammatory drugs (NSAIDs), or stress in animal models [6]. In the same way, various antioxidant enzymes such as superoxide dismutase (SOD), an important radical superoxide scavenger, and Glutathione peroxidase (GSH-Px), an enzyme involved in the elimination of hydrogen peroxide and lipid hydroperoxides, play an important role in cell protection [7, 8].

Recently, the role of neutrophils in the gastric lesions induced by NSAIDs, acetic acid, or ethanol has been pointed out [9, 10]. These leukocytes adhere to endothelial cells, thereby blocking capillaries and inducing damage to the endothelial cells through the release of proteases, leukotrienes, and active oxidants [11, 12].

Taking into account that lipid oxidation products may exert anti-inflammatory properties [13, 14], the aims of this study were to determine whether the treatment with OSO (4, 12, and 24 mg/kg) might reduce acute gastric ulceration induced by absolute ethanol and if it is so, to determine the potential changes in the activities of certain antioxidant enzymes such as SOD, catalase (CAT), and GSH-Px. Thiobarbituric acid reactive substances (TBARS) were also measured.

## 2. MATERIALS AND METHODS

### 2.1. Chemicals

All reagents used for determinations of SOD, CAT, GSH-Px, and TBARS were purchased from Sigma Chemicals (St. Louis, Mo,USA). Other reagents of analytical grade were obtained from normal commercial sources.

### 2.2. Animals and treatments

Male Sprague/Dawley rats (180–200 g) were purchased from National Center for Laboratory Animal Production (CENPALAB, Havana, Cuba). They were randomly assigned to six groups. The animals were housed in macrolon cages (Tecniplast, Italy), in a standard bioclean animal room, and kept under a 12-hour light-dark cycle at 22–24°C and humidity 70–75%.

The animals were deprived of food for 24 hours before the experiments but had free access to water, and were allowed to acclimatize for one week before the experiment. All of them were carried out in accordance with the ethical guidelines for investigations with laboratory animals and were approved by Ethical Committee for Animal Experimentation of National Center for Scientific Research, Havana, Cuba.

OSO was administered intragastrically by a cannula, and was applied during four days (one daily) to each group. The doses of OSO applied for groups were (4, 12, and 24 mg/kg). Control rats received the vehicle orally (sunflower oil 0.12 mg/kg). Standardization of the preparation of OSO was carried out according to the following parameters: peroxide index (PI), which indicates the quantity of peroxide available in OSO, was 650 mmol/kg; iodine index, which is a measure of the unsaturation rate of OSO, was between 50 and 90 units; viscosity, which is a measure of the polymerization by condensation of the peroxides forming in OSO, was between 100 and 450 mPa·s.

Ulceration was induced as described by Robert [15] instilling absolute ethanol (1 mL/200 g body weight). OSO was administered 1 hour before the administration of ethanol. One hour after the experimental period, the animals were euthanized using ether overdose, and their stomachs were removed and opened along the greater curvature, and their lesions were examined macroscopically.

### 2.3. Ulceration index

The length and width of each lesion were measured by stereoscopy (Carl Zeiss, Berlin, Germany) and the sum of the products was expressed in terms of the ulcer index (UI, square millimeters). The measurement of ulcer index was determined by protocol-blinded researcher.

The gastric mucosa was scraped with glass slides and frozen at −20°C, for subsequent biochemical determinations.

### 2.4. Biochemical assays

Gastric mucosa was weighted and homogenized in 10% w/v of a solution of KCl 100 mM with EDTA 0.3 mM for TBARS, GSH-Px, and SOD, using a tissue homogenator Ultraturrax T25 Polytron at 4°C. Gastric mucosa homogenates for CAT enzymatic assay were obtained with a 50-mM phosphate buffer (pH 7) containing 1% Triton X-100 (1 : 9 w/v). The homogenates were centrifuged at 600 g for 60 minutes at 4°C and the supernatants were taken for biochemical analysis.

### 2.5. Determination of TBARS content

The levels of TBARS in the gastric mucosa taken as lipid peroxides index (LP) were measured according to a method described by Ohkawa et al. [16] with minor modifications. Briefly the homogenate was supplemented with 8.1% sodium dodecyl sulphate (SDS), 20% acetic acid, and 0.8% Thiobarbituric acid (TBA), and boiled at 100°C for 1 hour. After cooling, the reactants were supplemented with 2.5 ml n-butanol-pyridine (15 : 1) mixture, shaken vigorously for 1 minute, and centrifuged for 10 minutes. Absorbance was measured at 532 nm and the results were expressed as nmol of TBA per gram of proteins.

### 2.6. Determination of SOD activity

SOD activity was determined by the modified version from the method of Minami and Yoshikawa [17]. Briefly, fifty microliters of mucosa homogenate were mixed with 450 *μ*L of cold deionized water, 125 *μ*L of chloroform, and 250 *μ*L of ethanol. The mixture was centrifuged at 8000 g for 2 minutes at 4°C. Five hundred microliters of the extract were added to the reaction mixture containing 500 *μ*L of 72.4 mM tris-cacodylate buffer with 3.5 mM diethylene pentaacetic acid (pH 8.2), 100 *μ*L of 16% Triton X-100, and 250 *μ*L of 0.9 mM nitroblue tetrazolium (NBT). The reaction mixture was incubated for 5 minutes at 37°C before adding 10 *μ*L of 9 mM of pyrogallol dissolved in 10 mM HCl. Then, it was incubated for exactly 5 minutes at 37°C. The reaction was stopped with the addition of 300 *μ*L of 2 M formic buffer (pH 3.5) containing 16% Triton X-100. The absorbance was measured at 540 nm in a spectrophotometer. One unit of SOD enzymatic activity is equal to the amount of enzyme that diminishes the initial absorbance of nitroblue tetrazolium by 50%.

### 2.7. Determination of GSH-Px activity

Glutathione peroxidase was measured using a modified version of the method of Faraji et al. [18]. All reaction mixtures were dissolved in 20 mM sodium phosphate buffer containing 6 mM EDTA (pH 7.0). The reaction mixture consisted of 98.8 *μ*L of phosphate buffer, 700 *μ*L of 2.86 mM GSH, 100 *μ*L of 1 mM sodium azide, 100 *μ*L of 1 mM NADPH, and 4.2 *μ*L of GSH reductase (0.5 unit). Then, 10 *μ*L of the tissue homogenate supernatant were added to the reaction mixture and incubated at room temperature for 10–15 minutes. Afterward, 10 *μ*L of 30 mM t-butyl hydroperoxide dissolved in bidistilled water were added to the reaction mixture and measured at 340 nm for 7 minutes in the spectrophotometer. A molar extinction coefficient of 6.22×10^3^ *μ*mol was used to determine the activity of GSH-Px. The enzyme activity was expressed as international units of enzymatic activity/mg of protein. International units are expressed as *μ* moles of hydroperoxides transformed /min/mL of enzyme.

### 2.8. Determination of CAT activity

CAT was determined according to the method of Rice Evans and Diplock [19]. Homogenate of rat gastric mucosa was diluted with buffer, as described before, in order to obtain an adequate dilution of the enzyme. Then, 2 mL of the enzyme dilution were added to the cuvette and mixed with 1 mL of 30 mM H_2_O_2_, measuring the absorbance at 240 nm for 100 seconds. Initial absorbance of the reaction mixture must be around 0.5. The enzyme activity is expressed as the first-order constant that describes the decomposition of H_2_O_2_ at room temperature.

### 2.9. Protein assay

Protein concentrations were determined by the method of Lowry et al. [20] using bovine serum albumin as standard.

### 2.10. Histological evaluation of gastric mucosa

The samples of the gastric mucosa were taken from rats treated with OSO and compared with those taken from rats treated with ethanol. The gastric mucosa tissue was fixed in 10% formalin, then embedded in paraffin, and finally stained with hematoxylin and eosin (H&E). The histological study was performed using a light microscope, and it was performed by a pathologist blinded to the treatment protocol.

### 2.11. Statistical analysis

Data were expressed as means ± SEM and analyzed statistically using Kruskall Wallis test followed by Mann Whitney test which was applied for the rest of the markers. The 0.05 level of probability was used as statistical significance.

## 3. RESULTS

In Table 1, the protective effects of OSO on ethanol-induced gastric lesions are shown. Oral administration of absolute ethanol (1 mL/200 g body weight) induced multiple, elongated, reddish bands of hemorrhagic erosions in rat gastric mucosa. The ulcer index was 115.75± 20.29 mm^2^. In this experimental model, oral pretreatment with OSO before ethanol administration prevented ulceration. The UI (3 ± 2.27 mm^2^) was significantly lower than in the rats receiving ethanol alone.

Using TBARS content in the gastric mucosa as an index, lipid peroxidation was significantly increased (*P* < .05) from basal concentration of 0.038 ± 0.06 nmol/g of protein to 0.55 ± 0.20 nmol/g of protein after administration of ethanol. OSO did not reduce significantly the levels of TBARS in the gastric mucosa (Table 2).

Table 2 also shows that ethanol induced a remarkable and significant decrease of GSH-Px activity in rat gastric mucosa, whereas OSO induced a significant reversion of ethanol effect on this enzyme.

In similar manner, SOD activity was significantly decreased in gastric mucosa after absolute ethanol treatment, but in the rats pretreated with OSO, a significant reversion of SOD activity was observed especially with greater doses of OSO (Table 3).

In contrast, neither OSO nor ethanol induced significant changes in CAT activity (Table 3).

**Figure 1:**
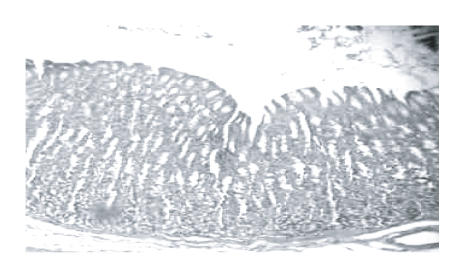
Normal histological structure of rat gastric mucosa.

**Figure 2:**
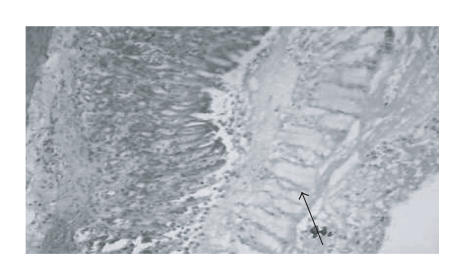
Histological appearance of the gastric ulcers 1 hour after ethanol treatment. Severe erosion with necrosis of gastric mucosa, detachment of necrotic gastric mucosa (arrow) H&E magnification, X 250.

**Figure 3:**
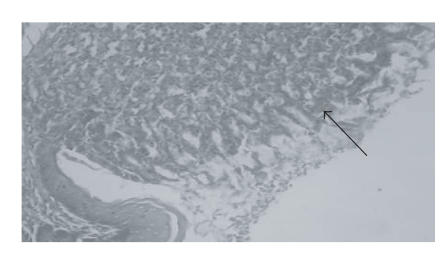
Rat pretreated with OSO, slight erosion of the gastric mucosa is observed. Arrow: H&E; magnification, X 100.

Figure 1 shows a normal histological structure of rat gastric mucosa. Figure 2 shows the histopathological injury in the rat gastric mucosa 1 hour after treatment with ethanol. OSO pretreatment reduced necrosis induced by ethanol (Figure 3).

## 4. DISCUSSION

Available data suggest that ROS plays a main role in tissue injury during the pathogenesis of various disorders of the digestive tract [21]. Oral administration of absolute ethanol in rats is noxious for the stomach, affecting the gastric mucosa topically by disrupting its barrier and provoking pronounced microvascular changes in few minutes after its application. Thus, rapid and strong vasoconstriction is accompanied by rapid and vigorous arteriolar dilation and this combination of microvascular events induces damage in mucosal capillaries [22, 23]. Currently, there is consensus that the former deleterious effects of ethanol on gastric mucosa are consequence of enhanced lipid peroxidation and decreased glutathione (GSH) levels. The involvement of oxygen radicals in ethanol-induced gastric injury was confirmed in cultured mucosal cells. Exposure to ethanol increased, in a dose-dependent manner, the generation of superoxide anions and the extent of cellular damage [24]. Salim [5] and Brzozowski et al. [25] have demonstrated that ethanol induces mucosal damage and impairs healing of lesions.

Our findings demonstrated that ethanol increases lipid peroxidation with respect to nontreated control rats, but no significant differences were found with respect to OSO- treated rats. It may be due to the presence of some aldehydes and hydroperoxides in OSO which might contribute to increase TBARS content in rat gastric mucosa.

The results also revealed a decrease of GSH-Px activity in gastric mucosa after ethanol treatment. GSH-Px is an important enzyme which plays a key role in the elimination of hydrogen peroxide and lipid hydroperoxides in the gastric mucosa cells [8].

In contrast with SOD and GSH-Px activities, which were significantly increased in rats treated with OSO (Tables 2 and 3), CAT activity was not significantly modified by treatments neither with ethanol nor with OSO (Table 3). This finding seems to be due to the fact that GSH-Px plays a much greater role than CAT in the removal of low steady-state concentrations of H_2_O_2_. Therefore, it seems that GSH-Px is the main antioxidant enzyme to remove H_2_O_2_ and CAT shows a lower affinity for that ROS. In this context, our result is in concordance with that reported by Billici et al. [26] and Kanter et al. [27].

The antioxidant activity of GSH-Px is coupled with the oxidation of reduced glutathione (GSH), which can subsequently be reduced by glutathione reductase with NADPH as reducing agent. Thus, inhibition of this enzyme may result in the accumulation of H_2_O_2_ with subsequent oxidation of lipids.

In contrast, OSO pretreatment 4, 12, 24 mg/kg induced a significant increase in GSH-Px activity after ethanol administration. This enhancement of GSH-Px activity suggests that the antiulcerogenic effect of OSO may be connected with GSH metabolism.

In summary, our results suggest that the gastroprotective effect of OSO in the rat gastric mucosal injury induced by ethanol might be mediated at least partially by its stimulant effect on antioxidant enzymes such as SOD and GSH-Px which constitute endogenous scavengers of ROS.

## Figures and Tables

**Table 1:** Gastric ulcer index (UI, mm^2^) in rats treated with absolute ethanol using different doses of OSO and the percentage of reduction of the lesions. UI was measured by stereoscopy (Carl Zeiss, Berlin, Germany) and the sum of the products was expressed in terms of the ulcer index (UI, Square millimeters) and % of reduction. The results are the means ± s.e.m. of five stomachs samples.

Groups	UI (mm^2^)	Reduction (%)

Nontreated control	0	—
Ethanol (1 mL/200 g)	115.75 ± 20.29	—
Sunflower oil (0.12 mg/kg) and ethanol	115 ± 11.62	0
OSO (4 mg/kg) and ethanol	18.5 ± 5.65*	84.01
OSO (12 mg/kg) and ethanol	7 ± 5.45*	93.95
OSO (24 mg/kg) and ethanol	3 ± 2.27*	97.40

*P < .05 compared with group treated with ethanol alone.

**Table 2:** Thiobarbituric acid-reactive substances (TBARS) and glutathione peroxidase (GSH-Px) in gastric mucosal damage induced by ethanol and pretreated with OSO. Data are expressed as nmol/mg prot of TBARS. The results are the means ± s.e.m. of five stomachs samples.

Groups	TBARS (nmol/g of protein)	GSH-Px (UI/g of protein)

Non treated control	0.038 ± 0.06	423.55 ± 65.96
Ethanol (1 mL/200 g)	0.551 ± 0.20	182.49 ± 32.16
Sunflower oil (0.12 mg/kg) and ethanol	0.539 ± 0.28	373.795 ± 85.74
OSO (4 mg/kg) and ethanol	0.515 ± 0.21	463.06 ± 63.06*
OSO (12 mg/kg) and ethanol	0.509 ± 0.18	440.018 ± 49.13*
OSO (24 mg/kg) and ethanol	0.506 ± 0.04	396.286 ± 36.43*

*P < .05 compared with group treated with ethanol alone.

**Table 3:** Superoxide dismutase (SOD) and catalase (CAT) activities on gastric mucosal damage induced by ethanol and pretreated with OSO. The results are the means ± s.e.m. of five animals per group. CAT activity is described as the first-order constant of the decomposition of H_2_O_2_ at 25°C/g of wet tissue. k_15_ is the constant kinetic of the first-order that describes the decomposition of H_2_O_2_ at room temperature. SOD activity is expressed in units of enzymatic activity/mg of proteins.

Groups	SOD (U/g of protein)	CAT (k_15_/g of tissue)

Non treated control	61.14 ± 6.13	4.83 ± 1.09
Ethanol (1 mL/200 g)	40.08 ± 5.29	5.174 ± 0.54
Sunflower oil (0.12 mg/kg) and ethanol	58.91 ± 7.71	4.260 ± 1.37
OSO (4 mg/kg) and ethanol	50.28 ± 22.94	3.906 ± 0.34
OSO (12 mg/kg) and ethanol	87.73 ± 17.78*	3.716 ± 0.67
OSO (24 mg/kg) and ethanol	70.01 ± 9.28*	4.862 ± 0.72

*P < .05 compared with group treated with ethanol alone.

## References

[1] Sechi LA, Lezcano I, Nuñez N (2001). Antibacterial activity of ozonized sunflower oil (Oleozon). *Journal of Applied Microbiology*.

[2] Remigio A, González YA, Zamora Z, Moleiro J (1998). Genotoxic evaluation of Oleozon by micronucleus assays in bone marrow and peripheral
blood in mice. *Revista CENIC, Ciencias Biológicas*.

[3] Zamora ZR, Torres D, Bouza M, Hernández D, Hernández F (2006). Oral Oleozon^®^, is an effective treatment in experimental giardiasis. *Revista CENIC Ciencias Biológicas*.

[4] Kvietys PR, Twohig B, Danzell J, Specian RD (1990). Ethanol-induced injury to the rat gastric mucosa. Role of neutrophils and xanthine oxidase-derived radicals. *Gastroenterology*.

[5] Salim AS (1990). Removing oxygen-derived free radicals stimulates healing of ethanol-induced erosive gastritis in the rat. *Digestion*.

[6] Szabo S, Pihan G, Dupuy D, Szabo S, Mozik G (1987). The biochemical pharmacology of sulfhydryl compounds in gastric mucosal injury and protection. *New Pharmacology of Ulcer Disease*.

[7] Halliwell B (1991). Reactive oxygen species in living systems: source, biochemistry, and role in human disease. *The American Journal of Medicine*.

[8] Halliwell B, Gutteridge JMC, Cross CE (1992). Free radicals, antioxidants, and human disease: where are we now?. *Journal of Laboratory and Clinical Medicine*.

[9] Wallace JL, Kennan CM, Granger ON (1990). Gastric ulceration induced by non-steroidal anti-inflammatory drugs is a neutrophil
dependent process. *American Journal of Physiology: Gastrointestinal and Liver Physiology*.

[10] Motilva V, Martin MJ, Luque MI, Alarcón de la Lastra C (1996). Role of polymorphonuclear leukocytes and oxygen-derived free radicals in chronic gastric lesion induced by acetic acid in rat. *General Pharmacology*.

[11] Jacobson ED (1992). Circulatory mechanisms of gastric mucosal damage and protection. *Gastroenterology*.

[12] Wallace JL, Chin BC (1997). Inflammatory mediators in gastrointestinal defense and injury. *Proceedings of the Society for Experimental Biology and Medicine*.

[13] Kochkov VN, Leitinger N (2003). Anti-inflammatory properties of lipid oxidation products. *Journal of Molecular Medicine*.

[14] Leitinger N (2003). Oxidized phospholipids as modulators of inflammation in atherosclerosis. *Current Opinion in Lipidology*.

[15] Robert A (1979). Cytoprotection by prostaglandins. *Gastroenterology*.

[16] Ohkawa H, Ohishi N, Yagi K (1979). Assay for lipid peroxides in animal tissues by thiobarbituric acid reaction. *Analytical Biochemistry*.

[17] Minami M, Yoshikawa H (1979). A simplified assay method of superoxide dismutase activity for clinical use. *Clinica Chimica Acta*.

[18] Faraji B, Kang HK, Valentine JL (1987). Methods compared for determining glutathione peroxidase activity in blood. *Clinical Chemistry*.

[19] Rice Evans C, Diplock AT, Burtin RH, Knippenberg PH (1991). Laboratory techniques in biochemistry and molecular biology. *Techniques in Free Radical Research*.

[20] Lowry OH, Rosebrough NJ, Farr AL, Randall RJ (1951). Protein measurement with the folin phenol reagent. *Journal of Biological Chemistry*.

[21] La Casa C, Villegas I, Alarcón de la Lastra C, Motilva V, Martín Calero MJ (2000). Evidence for protective and antioxidant properties of rutin, a natural flavone, against ethanol induced gastric lesions. *Journal of Ethnopharmacology*.

[22] Ko JKS, Cho CH, Ogle CW (1994). The vagus nerve and its non-cholinergic mechanism in the modulation of ethanol-induced gastric mucosal damage in rats. *Journal of Pharmacy and Pharmacology*.

[23] Glavin GB, Szabo S (1992). Experimental gastric mucosal injury: laboratory models reveal mechanisms of pathogenesis and new therapeutic strategies. *FASEB Journal*.

[24] Nordmann R, Ribiere C, Rouach H (1992). Implication of free radical mechanisms in ethanol-induced cellular injury. *Free Radical Biology and Medicine*.

[25] Brzozowski T, Konturek PC, Konturek SJ (1997). The role of melatonin and L-tryptophan in prevention of acute gastric lesions induced by stress, ethanol, ischemia, and aspirin. *Journal of Pineal Research*.

[26] Billici D, Süleyman H, Banoǧlu ZN (2002). Melatonin prevents ethanol-induced gastric mucosal damage possibly due to its antioxidant effect. *Digestive Diseases and Sciences*.

[27] Kanter M, Demir H, Karakaya C, Ozbek H (2005). Gastroprotective activity of Nigella sativa L oil and its constituent, thymoquinone against acute alcohol-induced gastric mucosal injury in rats. *World Journal of Gastroenterology*.

